# Evolutionary Algorithm-Based Complete Coverage Path Planning for Tetriamond Tiling Robots

**DOI:** 10.3390/s20020445

**Published:** 2020-01-13

**Authors:** Anh Vu Le, Nguyen Huu Khanh Nhan, Rajesh Elara Mohan

**Affiliations:** 1 ROAR Lab, Engineering Product Development, Singapore University of Technology and Design, Singapore 487372, Singapore; leanhvu@tdtu.edu.vn (A.V.L.); rajeshelara@sutd.edu.sg (R.E.M.); 2 Optoelectronics Research Group, Faculty of Electrical and Electronics Engineering, Ton Duc Thang University, Ho Chi Minh City 700000, Vietnam

**Keywords:** reconfigurable robot, cleaning robot, navigation planning, area coverage, energy aware, evolutionary algorithm

## Abstract

Tiling robots with fixed morphology face major challenges in terms of covering the cleaning area and generating the optimal trajectory during navigation. Developing a self-reconfigurable autonomous robot is a probable solution to these issues, as it adapts various forms and accesses narrow spaces during navigation. The total navigation energy includes the energy expenditure during locomotion and the shape-shifting of the platform. Thus, during motion planning, the optimal navigation sequence of a self-reconfigurable robot must include the components of the navigation energy and the area coverage. This paper addresses the framework to generate an optimal navigation path for reconfigurable cleaning robots made of tetriamonds. During formulation, the cleaning environment is filled with various tiling patterns of the tetriamond-based robot, and each tiling pattern is addressed by a waypoint. The objective is to minimize the amount of shape-shifting needed to fill the workspace. The energy cost function is formulated based on the travel distance between waypoints, which considers the platform locomotion inside the workspace. The objective function is optimized based on evolutionary algorithms such as the genetic algorithm (GA) and ant colony optimization (ACO) of the traveling salesman problem (TSP) and estimates the shortest path that connects all waypoints. The proposed path planning technique can be extended to other polyamond-based reconfigurable robots.

## 1. Introduction

Residential robots that help in everyday, dull, dirty, and routine cleaning tasks have recently gained attention. A recent survey from the international federation robotics states that, in 2020, floor cleaning robots are expected to be sold twice as much as the previous year [1]. These robots will become standard cleaning tools soon, with the clearing robot market predicted to reach USD 4.34 billion by 2023 [2].

A typical tiling robot consists of various mechanical and electronic components that help to perform autonomous operations [3]. In recent advancements, robots are housed with LiDAR sensors and wheel encoders in order to enable the SLAM ability [4]. With the new upgrades in these robots, various coverage path planning techniques have been explored. These path planning approaches should be able to understand the operating environment and respond adaptively [5]. The proper integration of sensor and perception units with path planning algorithms determines robot performance [6,7]. Most complete coverage path planning (CCPP) strategies focus on specific parameters to maximize efficiency such as successful navigation or path following, obstacle avoidance, minimum distance traveled, and energy consumed. Concerning cleaning robots, the path planning algorithm concentrates on establishing maximum area coverage with less area recovered.

CCPP algorithms have mostly been constructed for workspace modeling methods. These widely used approaches include the exact cellular-based decomposition method [8], Morse-based cellular decomposition [9], graph theory-based coverage [10], landmark topological-based coverage [11], and 3D coverage [12,13]. Among the methods studied, the most popular was the approximate cellular decomposition proposed by Choset [14] due to its flexibility in adapting to distinct scenarios. This algorithm decomposes the generated grid map into smaller sub-maps. Next, according to the obstacle density in each sub-map, the algorithm generates a navigation trajectory that can cover the entire area [15,16]. In approximation decomposition, researchers have proposed various alternative grid designs to improve the performance of the robot, such as grid-based decomposition [17], a wavefront-based algorithm [18], a neural network-based algorithm [19], and a hexagonal, spanning tree-based method [20].

However, the proposed coverage algorithms in previous literature were developed and tested extensively on robots with a fixed morphological design facing coverage issues when accessing constrained and narrow spaces. The waver design was introduced by Prabhakaran et al., where a cleaning robot called hTetro would change its morphology based on the perceived environment [21,22]. Since its re-configuring ability and its interaction between obstacles were different from traditional robots, the existing coverage algorithms were not suitable for it. In another work [23], we proposed optimal path planning for tiling coverage. This work focused on the TSP-based route selection form the generated way-points with respect to the energy cost. Although the TSP-based CCPP algorithm could optimize routes to reduce the energy cost, the computational complexity of the algorithm is significant. Such an algorithm is still considered an NP-hard problem, even with simple work spaces with no obstacles [24]. Especially when there is a long queue of navigational sequences (N(N−1)!)/2 for *N*, it is a challenging task to generate optimal solutions with TSP. Simple techniques such as spiral, greedy, and zigzag path patterns were implemented in a conventional cleaning robot with a formulated TSP [25]. At that time, the advanced strategy in this field of research was to apply GA [26] and ACO [27]. These evolutionary-based approaches were constructed under a collective learning process througha population of individuals [28]. By continuously running processes of mutation, recombination, and selection, the algorithm could generate optimal solutions rapidly, even in a larger workspace.

In this paper, we present the design of a novel reconfigurable robot called hTetrakis with three diamond-based modules that are able to change the morphology to overcome the constraints of environmental settings. Moreover, due to the reconfigurability of this hTetrakis robot, the energy cost function during navigation takes the activities of reconfiguration and locomotion, including translation, transformation, and orientation correction, into account to yield more precise results. The main contribution of this paper is twofold. First, we define a CCPP framework for reconfigurable tiling robots. Second, we construct the TSP cost function based on the energy profile of each action of the hTetrakis robot to derive the sub-optimal trajectory in the working environment before the robot conducts the complete coverage navigation.

The outline of this paper is presented as follows. Section 2 details the robot hTetratiks architecture. Section 3 describes how to represent hTetrakis on the grid workspace. The CCPP framework for the tiling robot by the tiling theory is given in Section 4. The optimal CCPP of the proposed method is evaluated in the experimental result section (Section 5), and the conclusion and the future works are discussed in Section 6.

## 2. Architecture of the hTetrakis Robot

**Figure 1:**
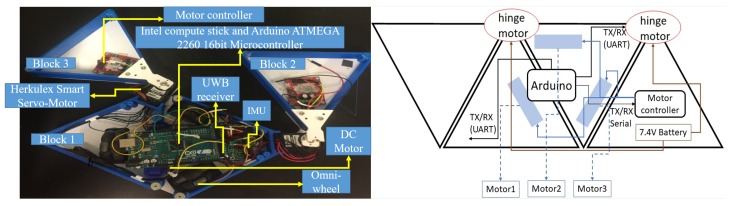
Electronic components of hTetrakis.

**Figure 2:**
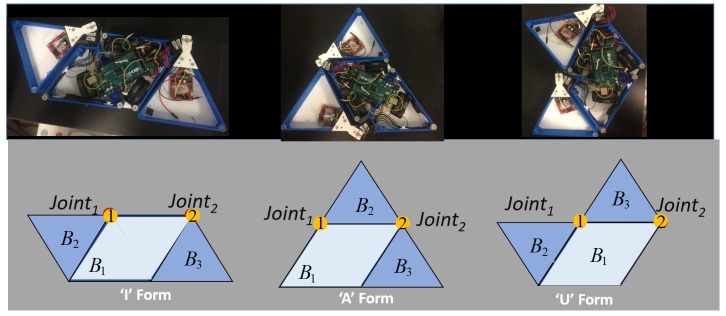
hTetrakis reconfiguration.

The design principle of the hTetrakis platform is based on the polyiamond hinge dissection principle. The building block of this platform is an equilateral triangular block, called a moniamond. There are four moniamonds in the platform, out of which the inner moniamonds are connected to form a trapezoidal block. All moniamond blocks are connected by a planar revolute joint, shown in Figure 1. When the moniamonds are rotated about the hinged joint, the platform attains three distinct configurations, called A, I, and U, as shown in Figure 2. The trapezoidal block is mounted on three Omni wheels with a diameter of 60 mm. Each omnidirectional wheel is driven by a DC motor with a gear ratio of 250:1, a 7.4 V voltage rating, a maximum torque of 769 Nmm, and a speed of 55 rpm. Each hinge joint consists of a Herkulex Drs 0201 servo motor with a voltage rating of 7.4 V and a standby stall torque of 24 kgcm. The outer monoiamond blocks in the I form are mounted on caster wheels that provide support during navigation. The outer structure of the robot platform and the motor housing in the hinge were fabricated and printed with polylactic acid (PLA) material using a Cubicon 3D printer. The base plate was fabricated using acrylic material using a laser cutter machine. A Lipo battery of 7.4 Volt and an Arduino Atmega 2560 16-Bit microcontroller are used to power the entire platform and control the locomotion and reconfiguration, respectively. A Bluetooth 4.0 module is used to communicate between the user and the microcontroller. The autonomy of the platform consists of an ultra-wideband (UWB)-based radio navigation system that monitors the position and the heading angle. The static UWB devices (transmitters) are kept in the workspace environment. Another UWB, called the receiver, is placed on the robot platform. The UWB system continuously estimates and updates the distance between the transmitters and the receiver. The autonomy system also consists of an on-board IMU that provides the heading angle (yaw rotation) of the platform. The platform locomotion follows the principle of the parallelogram law of vector addition applying on wheel velocities, and the resultant platform velocity is either translational along the X and Y linear direction or a zero-pivot.

## 3. hTetrakis Energy Workspace

### Representation of hTetrakis in a Workspace

**Figure 3:**
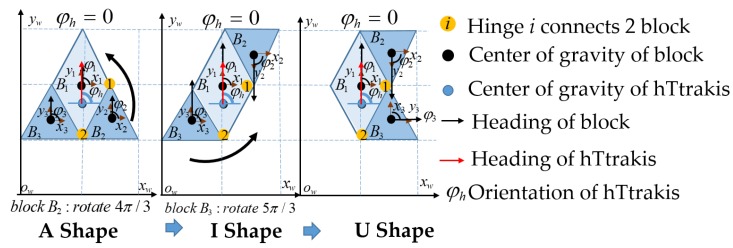
The hTetrakis robot inside the grid-based workspace and transformation processes from A to I and then to U.

The representation of the hTetrakis as the waypoint inside the predefined workspace is set at the robot center of mass (COM). Figure 3 describes the robot COM at global coordinates and the relation of the robot block’s local coordinates when the robot shifts the shape from an I to an A and then to a U. Specifically, the hTetrakis at waypoint *w* is denoted by the robot location $x_{h}^{w},y_{h}^{w}$ and robot orientation of block $B_{1}$ as $\varphi_{h}^{w}$. The robot block *i* is defined by $\left\{x_{i}^{w},y_{i}^{w},\varphi_{i}^{w}\right\}$, where *i* is the block number among three robot blocks $\left(i\in \left\{B_{1},B_{2},B_{3}\right\}\right)$ with robot block mass $m_{1},m_{2},m_{3}$ and the block length from COG $l_{b}$.

**Figure 4:**
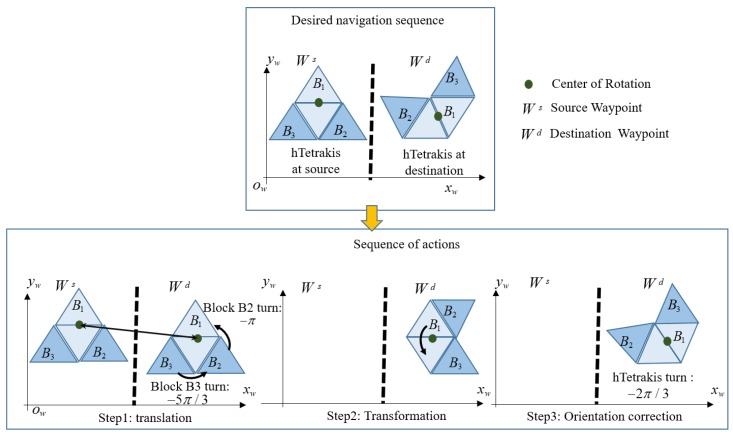
Sequence of action to navigate from a source waypoint $W^{s}$ to a destination waypoint $W^{d}$.

Figure 4 shows the sequences of actions when the robot navigates to connect a source waypoint $W^{s}$ and destination waypoint $W^{d}$. Specifically, hTetrakis performs three motions, including transformation, translation, and orientation correction to fit hTetrikis in a specific morphology within the workspace. The energy consumption during the navigation will be modeled from the travel distance of three actions multiplied by the corresponding mass of the robot blocks. The turning angle $\theta_{i}$ of the robot block to shift from one shape to another is shown in Table 1. Table 2 shows the tuning radius between the source and destination shapes. The orientation correction is the sum of the robot orientation at the destination location $\varphi_{h}^{d}$ and the orientation correction after shape-shifting $\varphi_{h}^{s}$. As described in Figure 4, the robot orientation after and before the transformation is kept stationary. The complete coverage path planning for the predefined workspace is described in the following section.

## 4. The Complete Coverage Path Planning (CCPP) Framework for hTetrakis by Tiling Theory

### 4.1. Tetriamond Tiling Theory for CCPP

**Figure 5:**
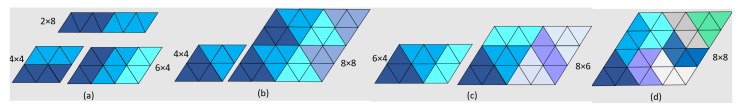
hTetrakis covers the workspace by tileset. (**a**) 2 × 8 workspace; (**b**) 4 × 4 workspace; (**c**) 6 × 4 workspace, (**d**) 8 × 8 workspace 8 × 8 worskpace.

The tiling theory addresses the tileset generation in the CCPP framework for the tetriamond-inspired, reconfigurable hTetrakis robot. By reordering the sides of the triangles along the various edges, we can obtain various configurations, shown in Figure 5. For instance, diamonds and triamonds have one configuration. However, tetriamonds, pentiamonds, and hexiamonds have 3, 4, and 12 configurations, respectively. The tetriamond has three distinct forms, i.e., I, A, and U. The following theorem is applied for hTerakis to tile a parallelogram *a*×*b*.

**Theorem** **1.** *Combinatorial Rules for Tetriamonds.*

*Any a×b can be tiled with all the three forms, i.e., the A, I, and U sets of tetriamonds, if (and only if) both a and b are multiples of 8.*

**Proof.**  A set of I, U, and A forms the smallest parallelogram of size 8 × 8 shown in Figure 5. Therefore, any parallelogram where the number of triangles is a multiple of 32 can be filled with all three forms. Let ‘a’ and ‘b’ be the number of triangles along both sides of the parallelogram to be tiled. The number of triangles (the area of the parallelogram) of this (a × b) parallelogram is ab/2. If (ab/2) = 32n (multiple of four), where n is a natural number, then this parallelogram can be filled with all three forms completely, without any void. The number of triangles along each side must be greater than eight. This confirms the formulation of the theorem that any parallelogram a × b can be tiled with all three forms entirely if and only if both a and b are multiples of 8. □

**Figure 6:**
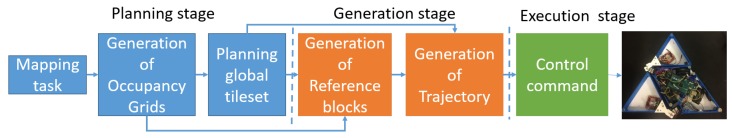
Flowchart for the complete coverage path planning (CCPP) robot motion planner for hTetrakis.

**Figure 7:**
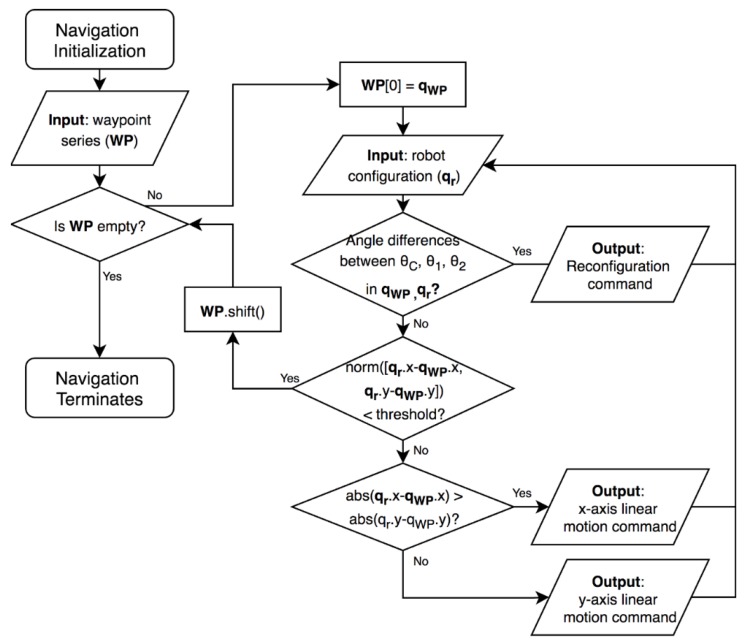
Flowchart of the robot motion planner for hTetrakis.

The process of the CCPP framework in Figure 6 consists of three stages: planning, generation, and execution state. Specifically, given the workplace that can be tiled completely with robot shapes among suggested tiling patterns A, U, and I by the above tiling theorem, the appropriate location and the orientation inside the workspace of the selected tile are determined by the backtracking algorithm [29]. After finishing identification for the considered tile, the backtracking algorithm tries to tile the rest of the tiling set in the workspace. If the next tile cannot be located by the algorithm, then the other possible placements are tried. Even after trying all possible placements, when the algorithm departs from tile combinations, then it backtracks to the previous tiling pattern and executes the same procedure with the new tiling pattern among the tileset. The same process continues until the considered workspace is tiled entirely. The navigation sequence by robot actions as described in Figure 4 derives the optimal energy consumption. The appropriate commands are made to control the manipulator modules of the robot to complete the optimal estimated sequence, as shown in Figure 7.

Once the navigation process initializes, the system will load the waypoint series *W* from the path planner and begin a waypoint clearing loop. Within the loop, the robot will be continuously updating its current configuration at waypoints based on the sensor readings. The algorithm prioritizes robot reconfigurations to robot linear motion, which implies that, if a discrepancy between the current robot morphology at the source waypoint $W^{s}$ and the next waypoint’s morphology at the destination waypoint source waypoint $W^{d}$ is found, it will issue a command to the hTetrakis microcontroller to perform a reconfiguration command by controlling the analog angle values sent to the servo motors. The algorithm then checks whether the robot’s current position $x_{h}^{w},y_{h}^{w}$ is close enough to the coordinates of the destination waypoint $W^{d}$. The waypoint will be considered as cleared if the distance difference is within an acceptable threshold range, and a new waypoint will be issued until WP is empty; otherwise, it will compare the distance difference between the two waypoints in the X and Y axes. The robot will then move along the axis that yields the larger absolute distance difference, eventually arriving at the position of the destination waypoint $W^{d}$. Similarly, the linear motion commands assigned during this process will be sent to the hTetrakis microcontroller, which will encode the commands into PWM values for the three wheels to move towards the desired direction.

### 4.2. Assigning the hTetrakis Blocks Location

**Figure 8:**
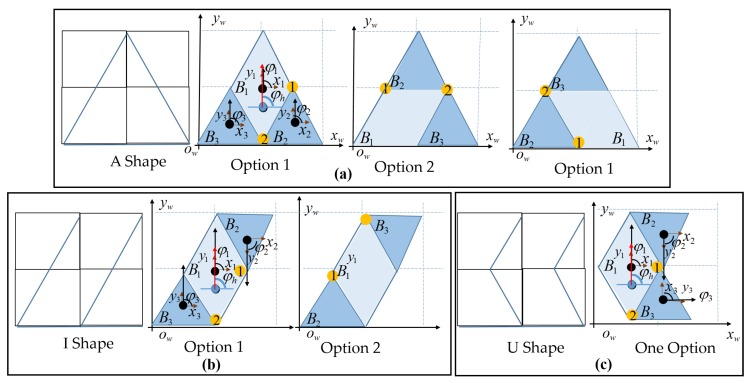
Block location options for symmetrical and asymmetrical shapes. (**a**) Symmetrical A; (**b**) Symmetrical I; (**c**) Asymmetrical U.

**Figure 9:**
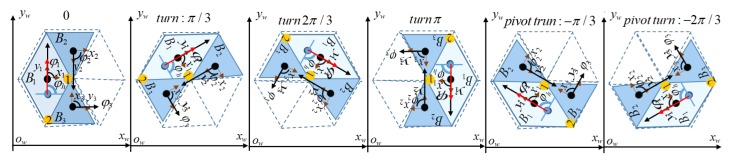
Robot heading with a clockwise turn and an anticlockwise pivot turn.

Tilesets by the tiling theory provide only the shape of the robot within the workspace. With one specific robot shape, the locations of blocks $B_{1},B_{2},B_{3}$ can arrange in different orders, and these options create a different robot COM and different navigation sequences within the workspace. The block order of the robot for the symmetric A and I shapes and the asymmetric U shape are denoted in Figure 8a,b and Figure 8c, respectively. Since the robot has two hinges to link blocks, the asymmetric U shape has only one block location as in Figure 8c. On the other hand, as shown in Figure 8a,b, the rotation of the hinge of symmetrical morphologies such as A and I issues several options for robot block locations. Furthermore, block location is also based on the robot orientation in the predefined workspace. The locations of the blocks with the robot heading for the A shape, for one predefined workspace with a size of ($w_{r},w_{c}$) and a tile with an appropriate tileset, are shown in Figure 9.

**Figure 10:**
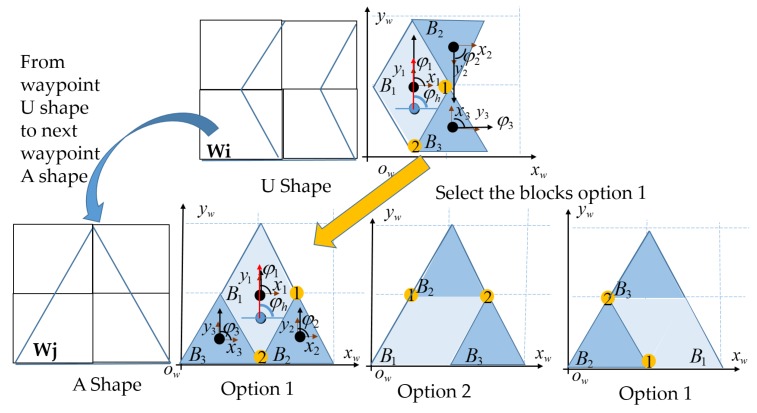
Robot block location when changing shape from U to A.

Algorithm 1 is used to locate the blocks for all robot shapes. The algorithm applies the loop to visit all tilesets to set each block’s pattern. If the current shape *t* is symmetric, such as A, and I, the block location of the tile *t* − 1 with the smallest orientation offset to the tile *t* is picked. As a sequence, the orientation is reduced because the orientation correction energy during robot navigation is minimized. Equation (6) derives the block locations for symmetrical tiles *t* with Ω options. Figure 10 shows an example of selecting a block with an A shape given a previous U shape. The algorithm selects the block locations of the A shape as shown in Figure 10a since it has the same heading as that of the previous U shape heading.

(1) $$\hat{t}=\underset{p\in \Omega }{argmin}\left(\right|\varphi_{h}^{t}-\varphi_{h}^{t-1}\left|\right)$$

**Algorithm 1:** block location setting**1Function** OPTIMAL BLOCKS{predefined workspace, selected tileset}:**2** define {$w(w_{r},w_{c})$}**3***i* ←1, *j* ←1, *t* ←1**4** **for all***i*, *i* ←1, do**5**  **for all***j*, *j* ←1, do**6**   **location**w(i,j) is COM of tile *t*:**7**    **if** tile *t* is asymmetrical tile:**8**     *Locate*: tile *t* blocks as Figure 8c**9**    **else if** tile *t* is symmetrical tile:**10**      *Execute:* shifting from t−1 to *t***11**      *Find:* blocks of tile having the similar heading with the heading after transforming**12**      *Locate*: tile *t* blocks as Figure 8a,b**13**    **end****14**   **end****15**  **end**
**End Function**


### 4.3. Optimal Navigation Sequence

The operation whereby a robot covers the workspace entirely with a predefined shape is separated into three independent steps: translation, transformation, and orientation correction, as shown in Figure 4. Specifically, to navigate from any waypoint $W^{s}\left(x,y\right)$ to the next waypoint $W^{d}\left(x,y\right)$ within the workspace, a robot will navigate linearly; for example, Block 1 of a robot (COR) will arrive at the destination waypoint position, then pivot around the COR to the correct orientation of the relevant shape in the workspace, and finally transform to the desired shape of the next waypoint. In this paper, the energy consumption of each operation during navigation of the robot is proportional to the summation of the linear moving distance and the mass of the robot. The translation energy is the 2D Euclidean distance displacements multiplied by the mass of the platform, as in Equation (2), which corresponds to all three blocks and navigates robot block 1 from the COR source waypoint to the COR destination waypoint. The energy of transformation is found by summing the rotations of $\phi_{2}$ of blocks 2 and $\phi_{3}$ of block 3 around the hinge of the robot, and it is then multiplied by the corresponding mass of the blocks $m_{i}$, as in Equation (3). The energy of orientation correction is the absolute rotation distance multiplied by the mass of all three blocks around the COR from the source direction $\theta_{s}$ to the destination direction $\theta_{d}$, as in Equation (4)

The costweight of pair *k* including source $W^{s}\left(x,y\right)$ and destination $W^{d}\left(x,y\right)$ waypoints are the linear summation of translation, transformation, and orientation correction operations, as shown in Equation (5).

(2) $$D_{tranl}=\sum_{i=1}^{3}m_{i}\sqrt{{\left(x_{i}^{s}-x_{i}^{d}\right)}^{2}+{\left(y_{i}^{s}-y_{i}^{d}\right)}^{2}}$$

(3) $$D_{tranf}=\left(m_{2}\phi_{2}+m_{3}\phi_{3}\right)l_{b}$$

(4) $$D_{ori}=\sum_{i=1}^{3}m_{i}l_{b}\left|\varphi_{h}^{d}-\varphi_{h}^{s}\right|$$

(5) $$C^{k}\left(W^{s},W^{d}\right)=D_{tranl}^{k}+D_{tranf}^{k}+D_{ori}^{k}$$

(6) $${\hat{\rho }}_{k}=\underset{k^{*}\in \Omega }{argmin}\sum C^{k*}\left(W^{s},W^{d}\right)$$

Finding the trajectory ρ linking all pairs of waypoints modeled in Equation (6) is the optimization problem of the classical TSP, which is an NP-hard problem. O(n!) represents the required steps to solve this problem for a brute force search. If the workspace is large, it performs extremely slowly and is not practical for real applications. To ease the complexity of this problem, evolutionary algorithms are applied. In this paper, a GA and ACO are used to solve the TSP of sequencing the navigation order. The works of [26,27] explain the GA and ACO in detail and how they are implemented to solve the TSP. The GA repeats the selection and reproduces steps to dismiss genes of poor performance after each iteration while preserving the useful information of genes in each generation. On the other hand, the ACO uses the stochastic approach to solve the TSP by varying the decisions of the ant agents at the nodes and by constantly updating the pheromones left on each path. Both GA ACO methods apply the meta-heuristic technique by gradually enhancing found solutions. The meta-heuristic technique does not guarantee that the solution of the navigation sequence is the optimal global solution; however, the GA and ACO methods are able to derive the almost optimal solutions and reduce running time significantly.

## 5. Experiment for the CCPP Framework

The experiments were conducted in a simulation environment to compare the costweights’ generated path and a real testbed environment to verify the energy consumption of CCPP.

### 5.1. Simulation Environment

**Figure 11:**
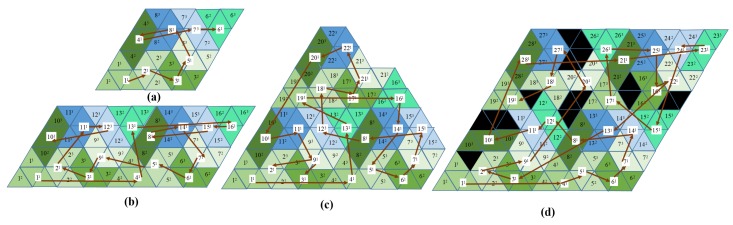
Optimal sequences by ACO for simulation workspace. (**a**) 4 × 4 workspace; (**b**) 4 × 8 workspace; (**c**) arbitrary workspace; (**d**) 8 × 8 workspace with obstacles.

Several workspace sizes in Matlab Simulink with and without obstacles were selected to evaluate the efficiency by the costweight of the generated path for tested methods: zigzag, spiral, greedy search, and the GA and ACO methods. The workspaces were divided into triangle shapes equal to the hTetrakis block. A workspace with a regular size, workspaces with an irregular size, and the workspaces with obstacles are Figure 11a,b, Figure 11c, and Figure 11d, respectively. Inside the simulated workspaces, the obstacle regions are denoted with black, which will be ignored when generating the tileset to fit the robot inside the workspace. Note that the I shape can cover a regular workspace completely and fails to completely cover an irregular workspace or a workspace with obstacles. The proposed tiling theory for the hTetrakis form is used to prove that the specific workspace can be filled by the robot’s available shapes, A, I, and U, and then by the backtracking algorithms [29] to derive the tileset solutions. The navigation sequences for all simulation workspaces of the ACO method yield the lowest costweight, as denoted by the red arrows in Figure 11. For the GA, mutation probability = 0.1, and chromosome = 100, and for the ACO, evaporation probability = 0.9, and the number of ants = 100. These values were the best results of 20 trials. Analysis for optimal parameter estimation will be performed in future work.

**Figure 12:**
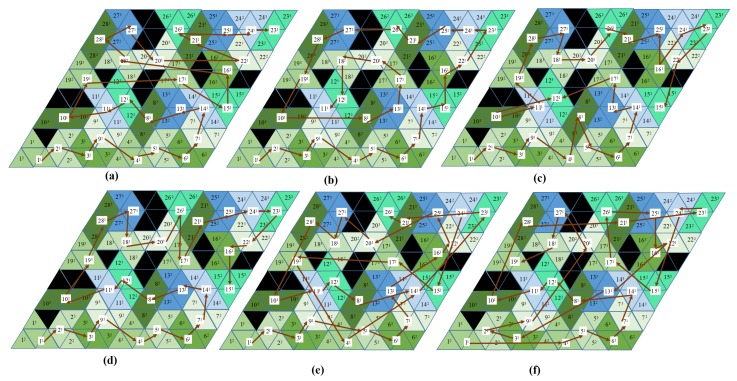
Trajectories of tested different methods. (**a**) Zigzag scanning; (**b**) spiral scanning; (**c**) random search; (**d**) greedy search; (**e**) GA; (**f**) ACO.

**Figure 13:**
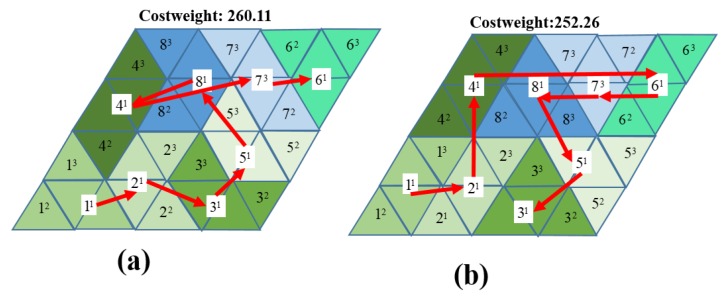
CCPP sequence of different tilesets of a 4 × 4 workspace. (**a**) Optimal sequence with A, I, and U; (**b**) optimal sequence with A and I.

The numerical comparisons in terms of the costweights and the execution time of the zigzag, spiral, random search with 1000 iterations, greedy search, and GA and ACO methods for the workspace in Figure 11d are shown in Table 3. The trajectories of tested different methods this workspace are shown in Figure 12. It is worth noting that zigzag and spiral methods connect the predefined waypoints in an orderly manner; on the other hand, the greedy search depends on the number of trials for an optimal solution. For this reason, the GA and ACO require less path generation time than the greedy search. The costweights of the ACO are the lowest among all test methods due to the optimization of the probability distribution. In Figure 12e,f, GA and ACO navigation sequences prefer the paths that connect two waypoints with the same morphology instead of linking the waypoint at the nearest locations. The trajectories of proposed method for A, I, U tileset and A, I tileset inside the workspace 4 × 4 are represented in Figure 13. From the results, if the neighboring tiles are the same as the current tile, the GA and ACO can select the next waypoint with less orientation correction. For example, the algorithm links Waypoint 3 to Waypoint 5 with zero degrees of orientation instead of Waypoint 2 with 4π/3. Furthermore, the GA and ACO algorithms select the shape transformation from A to I rather than from A to U, from U to I, or from U to A, since the robot rotates only one block with a value of 3π/4 to complete transformation. Since the amount of transformation and orientation correction is reduced when navigating between each pair of waypoints along the navigation sequence, the total costweight is lower, and the transformation times are minimized.

### 5.2. Real Environment Testbed

**Figure 14:**
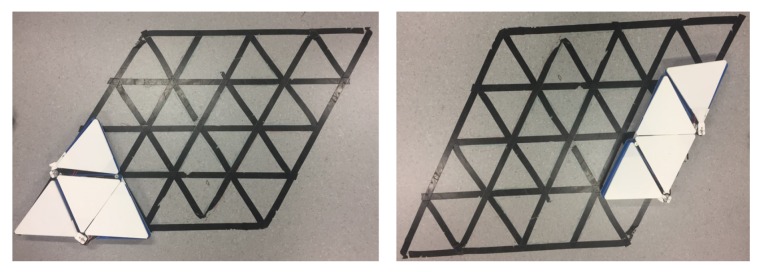
Realtestbed.

The costweights generated for the simulation workspaces in Figure 11a were validated for energy consumption efficiency in a real testbed environment as shown in in Figure 14. The robot navigated autonomously and in an orderly manner to clear all waypoints along the generated sequence. The robot localized itself inside the workspace by using the indoor localization UWB sensors. Power consumption of the robot was monitored through current sensors mounted at the robot battery. The sampling frequency of the measured current was 10 KHz at a 7 V voltage. The maximum motor speed was 50 rpm.

**Figure 15:**
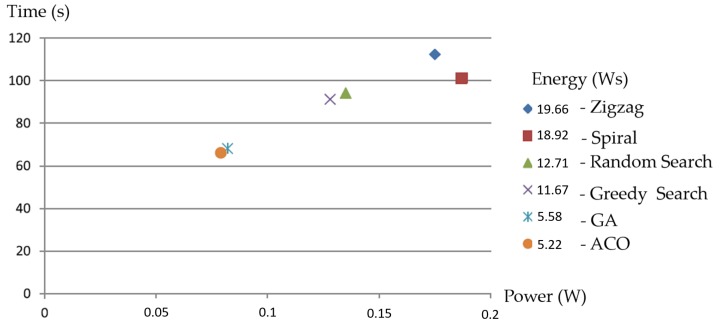
Energy consumption validation in a 4 × 4 real environment.

Figure 15 shows the energy consumption and travel time of all test methods for the real workspace. From the numerical values, we noticed that a smaller estimated costweight was matched with a smaller energy value. Specifically, the navigation sequence of zigzag yielded a higher value of energy consumption, and close behind was the spiral. The ACO and GA methods yielded both the least energy consumption and the least workspace coverage time. ACO was about 30% lower than the second-best method, i.e., the greedy search. These results show that the TSP with a heuristic-based evolutionary ACO or GA technique is a possible energy-aware CCPP technique for tiling robots in real environments.

Table 4 shows the energy for individual action among translation, rotation, and transformation that is used by the robot to finish the generated CCPP. Note that three DC motors at robot blocks need to rotate to drive the robot to the required location; however, to accomplish the rotation of elements, the servo motor at the robot hinge will rotate at an absolute value of either pi or 5pi/3, as described in Table 2. We can observe that the translation takes the most energy, the transformation stands at the second place, and the orientation consumes the least energy.

## 6. Conclusions

The proposed CCPP framework for the tetriamond, reconfigurable tiling robot has shown efficiency in generating the best navigation sequence among the tested methods in both simulation and real testbed environments. Modeling the energy as the travel distance is an effective method of estimating the energy. Optimizing the order of traveling sequences by the TSP is a feasible solution for the proposed robot platform to conduct complete workspace coverage. As such, this paper should be of interest to a broad readership, including those interested in sensor-based localization, energy preservation, complete coverage path planning, and reconfigurable tiling robotic systems. The issues of dynamic obstacles in working environments and modeling energy with consideration of friction is an interesting research topic for future work.

## Figures and Tables

**Table 1:** The hTetrakis block’s turning angles between the source and destination shapes.

	$W^{d}$	A Shape $B_{1}$ $B_{2}$ $B_{3}$	I Shape $B_{1}$ $B_{2}$ $B_{3}$	U Shape $B_{1}$ $B_{2}$ $B_{3}$
$W^{s}$				
A Shape		0 0 0	0 π 0	0 π 5π/3
I Shape		0 −π 0	0 0 0	0 0 5π/3
U shape		0 −π −5π/3	0 0 −5π/3	0 0 0

**Table 2:** The hTetrakis block’s tuning radii between source and destination shapes.

	$W^{d}$	A Shape $B_{1}$ $B_{2}$ $B_{3}$	I Shape $B_{1}$ $B_{2}$ $B_{3}$	U Shape $B_{1}$ $B_{2}$ $B_{3}$
$W^{s}$				
A Shape		0 0 0	0 $l_{1}$ 0	$l_{1}$$l_{1}$ 0
I Shape		0 $l_{1}$ 0	0 0 0	0 0 $l_{1}$
U shape		$l_{1}$$l_{1}$ 0	0 0 $l_{1}$	0 0 0

**Table 3:** Comparison of CCPP methods.

Method	Euclidean	Cost	Generation
	Distance	Weight	Time
Zigag	982.12	933.29	0.01
Sprial	978.21	951.18	0.05
Random search	952.52	836.22	27.14
Greedy search	943.32	819.21	29.15
Propsed method GA	958.38	725.26	1.38
Propsed method ACO	993.39	715.59	1.19

**Table 4:** Energy consumption for individual action.

Method	Cost	Total	Translation	Transformation	Orientation
-	Weight	Energy (Ws)	Energy (Ws)	Energy (Ws)	Energy (Ws)
Zigzag	321.15	19.66	10.39	6.35	2.92
Spiral	325.29	18.92	9.22	5.74	3.96
Random search	300.19	12.71	7.19	3.38	2.14
Greedy search	286.25	11.67	6.26	2.93	2.48
GA	267.12	5.58	3.01	1.61	0.96
ACO	260.11	5.22	2.91	1.39	0.92
